# Prevalence and associated factors of symptoms of depression, anxiety and stress in pregnant women in Mozambique: a cross-sectional study

**DOI:** 10.1186/s40359-026-05390-9

**Published:** 2026-08-19

**Authors:** Elisa Paulo Joaquim Barros, Afonso Mazine Tiago Fumo, Milton Armando Teresa Malai Moçambique

**Affiliations:** 1 Faculty of Medicine, Eduardo Mondlane University, Maputo, Mozambique; 2 Beira Central Hospital, Beira, Mozambique; 3 Catholic University of Mozambique, Beira, Mozambique; 4 Infulene Psychiatric Hospital, Maputo, Mozambique

**Keywords:** Pregnancy, Depression, Anxiety, Stress, DASS-21, Mozambique

## Abstract

**Background:**

Mental disorders represent a major public health burden worldwide, disproportionately affecting women in low- and middle-income countries. The perinatal period is a time of increased biological, psychological, and social vulnerability, heightening the risk of depressive, anxiety, and stress symptoms. Despite the high relevance, data on maternal mental health in Mozambique remain scarce.

**Methods:**

A cross-sectional study was conducted in two health centers in Maputo. Pregnant women aged ≥ 18 years were systematically recruited. Psychological symptoms were assessed using the DASS-21. Prevalence ratios for depressive, anxiety, and stress symptoms were estimated using Poisson regression with robust variance.

**Results:**

Clinically significant symptoms (moderate to extremely severe) were observed in 38.9% of participants for depression, 57.6% for anxiety, and 14.4% for stress. Additional factors significantly associated with symptoms included household composition, marital status, family history of mental illness, occupational status, religion, and family planning practices.

**Conclusions:**

The findings highlight substantial psychosocial and structural vulnerabilities among pregnant women in Maputo. Systematic screening integrated into prenatal care, policies addressing economic and occupational insecurity, and further research exploring protective factors and culturally relevant interventions are warranted.

## Background

Mental disorders represent a major global public health challenge, contributing substantially to years lived with disability, particularly among women in low- and middle-income countries [1]. The perinatal period is characterized by heightened biological, psychological, and social vulnerability, increasing susceptibility to depressive, anxiety, and stress symptoms [2, 3].

Untreated maternal psychological distress has been linked to adverse obstetric outcomes and negative child development [4]. Evidence from low- and middle-income countries suggests that social determinants, such as poverty, food insecurity, and limited support networks, further exacerbate maternal vulnerability [5, 6].

Beyond epidemiological indicators, contemporary perspectives conceptualize mental health not merely as the absence of disorder but as a multidimensional state involving adaptive functioning, emotional regulation, and well-being. A biopsychosocial framework highlights the interaction between biological predispositions, psychological processes, health behaviors, and social context in shaping maternal mental health outcomes [7, 8]. Positive psychological processes and health-related behaviours may contribute to emotional adaptation during pregnancy.

In Mozambique, mental health services remain limited, with shortages of trained professionals and insufficient integration into primary and maternal care [9, 10]. Systematic screening for perinatal psychological symptoms is not routinely implemented, representing a critical gap in evidence-based maternal health policy. Generating local data on prevalence and associated factors is essential to inform interventions, optimize clinical care, and guide public health strategies [11].

This study aimed to estimate the prevalence of depressive, anxiety, and stress symptoms and to identify sociodemographic and clinical factors associated with these outcomes among pregnant women attending two health centers in Maputo.

## Methods

### Study design

A cross-sectional, analytical study was conducted in May 2025 at two primary-level health centers in Maputo, Mozambique: Alto Maé (urban) and Zimpeto (peri-urban). These sites were selected for their comparable characteristics, differing primarily in location, allowing for contextual comparisons.

### Population and sample

Based on the average monthly number of pregnant women attending each center (Alto Maé: 300; Zimpeto: 256), a proportional systematic probabilistic sampling strategy was applied. A systematic sampling interval (k) was calculated based on the estimated number of eligible attendees at each health center. After a random starting point was established, every k-th eligible participant was invited to participate. In cases of refusal or ineligibility, the next eligible participant was approached to minimise potential selection bias. The high participation rate (91.6%) reduces the likelihood of significant non-response bias, although it cannot be entirely excluded.

The sample size was calculated using the formula for an infinite population, assuming a 95% confidence level, a 5% margin of error, and an expected prevalence of 50%. After finite population correction for the 556 pregnant women attending both health centers, a minimum sample of 227 participants was required. Of the 250 women systematically invited, 229 provided informed consent and were included in the final analysis (participation rate: 91.6%).

Eligible participants were pregnant women aged ≥ 18 years attending prenatal care at Alto Maé or Zimpeto Health Centers, excluding those with severe medical conditions or cognitive/linguistic limitations that could impair participation.

### Instruments

Sociodemographic and clinical data were collected using a structured questionnaire adapted from previous studies [2]. Psychological symptoms were assessed using the Portuguese version of the Depression, Anxiety and Stress Scale-21 (DASS-21) [12, 13], which assesses symptoms over the previous two weeks. The instrument comprises three subscales (depression, anxiety, and stress), each with seven items scored from 0 to 3; subscale totals were multiplied by two according to standard scoring procedures.

Symptoms were considered present when scores reached the moderate threshold or higher (depression ≥ 14; anxiety ≥ 10; stress ≥ 19), in accordance with the validated Portuguese DASS-21 classification [12]. This criterion is widely used in perinatal research and allows identification of clinically relevant psychological distress while ensuring comparability across epidemiological studies [13].

### Data collection

Individual interviews were conducted by trained health professionals in private rooms at the health centers and lasted approximately 20–25 min. Data were entered directly into a REDCap electronic database (Research Electronic Data Capture, version 5.30.5), which incorporates automated consistency checks to enhance data quality.

Participants with symptom levels classified as moderate to extremely severe were referred to on-site psychology or psychiatry services; follow-up was not included in the study.

### Data analysis

Data were analysed using IBM SPSS Statistics (version 27.0; IBM Corp., Armonk, NY, USA). Continuous variables were summarised as means and standard deviations or medians and interquartile ranges, depending on their distribution, while categorical variables were presented as frequencies and percentages. Associations between sociodemographic and clinical variables and the dichotomised outcomes of depression, anxiety, and stress were examined using bivariate tests according to the type and distribution of the variables. Differences in means were assessed using Student’s t-test, while the Mann–Whitney test was applied when normality assumptions were not met. Associations between categorical variables were evaluated using Pearson’s chi-square test or Fisher’s exact test when expected cell counts were small. For multinomial categorical variables with statistically significant associations, adjusted residual analysis was used to identify the categories contributing to the association. Variables with *p* < 0.10 in the bivariate analysis were included in Poisson regression models with robust variance to estimate prevalence ratios (PR) and their corresponding 95% confidence intervals (CI), as recommended for cross-sectional studies [14]. For categorical predictors, reference categories were defined a priori and are indicated in the regression tables. Statistical significance was set at *p* < 0.05 [14]. Internal consistency of the DASS-21 subscales was assessed using Cronbach’s α coefficient. To ensure model parsimony and avoid overfitting, a total of 9 variables were entered into the depression model, 11 into the anxiety model, and 8 into the stress model, based on their statistical significance in bivariate analysis (*p* < 0.10).

### Ethical considerations

Following the Declaration of Helsinki [15], this study was approved by the Institutional Committee of Bioethics for Health of the Faculty of Medicine and Central Hospital of Maputo (approval no. CIBSFM&HCM/151/2024), and all participants provided written informed consent, ensuring confidentiality, anonymity, and the right to withdraw.

## Results

### Sociodemographic characteristics

Among the 229 pregnant women included in the study, most were aged 20–34 years, with a mean age of 28.8 years (SD = 5.9). The majority were in their second trimester of pregnancy and had completed primary or secondary education. Regarding marital status, common-law unions and married women predominated, and approximately one-third of participants lived with their family of origin. The median number of pregnancies was 2 (P25-P75: 2–3), while the median number of current live births was 1 (P25-P75: 0–2). Additionally, 24.5% reported a history of miscarriage or stillbirth. Table 1 presents the detailed sociodemographic and clinical characteristics of the participants.

**Table 1:** Sociodemographic and clinical characteristics of pregnant women treated in Maputo, Mozambique (*n* = 229)

Sociodemographic and clinical characteristics	Category	Freq (*n*)	%
Occupation	Yes	145	64.7
	No	79	35.3
Education	None	1	0.4
	Primary	19	8.3
	Basic	113	49.3
	Secondary	72	31.4
	Bachelor’s Degree	24	10.5
Gestational trimester	1st	60	26.3
	2nd	113	49.6
	3rd	55	24.1
Family planning	Yes	71	31.0
	No	158	69.0
Religion	Atheist	1	0.4
	Catholic	46	20.1
	Muslim	27	11.8
	Spiritist	8	3.5
	Protestant	145	63.3
	Other	2	0.9
Marital status	Married	45	19.7
	Common-law marriage	70	30.6
	Single	114	49.8
Monthly income	< 10,000 mtn	157	68.6
	From 10,000 to 15,000 mtn	56	24.5
	From 15,000 to 20,000 mtn	10	4.4
	> 20,000 mtn	6	2.6
Devotes time to leisure activities	Never	38	16.6
	Rarely	125	54.6
	Occasionally	49	21.4
	Frequently	17	7.4
Sleep quality	Satisfactory	170	74.6
	Unsatisfactory	58	25.4
Undergoing long-term treatment	No	198	88.0
	Yes	27	12.0
Type of long-term treatment	ART	22	
	Hypertension	1	4.2
	ART + Hypertension	1	4.2
Follow-up with Mental health providers	None	216	94.3
	Psychologist	12	5.2
	Psychiatry technician/psychiatrist	1	0.4
Relatives who have been or are currently being treated by mental health providers	None	220	96.5
	Psychologist	7	3.1
	Psychiatry technician/psychiatrist	1	0.4

### Prevalence of symptoms and associated factors

The overall prevalence of psychological distress symptoms according to the DASS-21 was high among the evaluated pregnant women. Considering all severity levels, 59.4% presented depressive symptoms, 65.9% anxiety symptoms, and 24.9% stress symptoms. At clinical levels (moderate to extremely severe), prevalence was 38.9% for depression, 57.6% for anxiety, and 14.4% for stress (Table 2).

**Table 2:** Data on depressive, anxiety, and stress symptoms

Variables	Classification	Freq(*n* = 229)	%
Total Depression Score DASS-21	Normal	93	40.6
	Mild	47	20.5
	Moderate	62	27.1
	Severe	20	8.7
	Extremely Severe	7	3.1
Total Anxiety Score DASS-21	Normal	78	34.1
	Mild	19	8.3
	Moderate	50	21.8
	Severe	38	16.6
	Extremely Severe	44	19.2
Total Stress Score DASS-21	Normal	172	75.1
	Mild	24	10.5
	Moderate	25	10.9
	Severe	8	3.5
	Extremely Severe	0	0.0

In the bivariate analysis, a statistically significant association was observed between depressive symptoms and the household composition (*p* < 0.001), marital status (*p* = 0.032), and family history of mental illness (*p* < 0.001). For anxiety, associations with household composition (*p* = 0.004) and family history of mental illness (*p* < 0.001) stood out. For stress, a significant association was found with marital status (*p* = 0.021) and family history of mental illness (*p* = 0.017) as shown in Table 3.

**Table 3:** Bivariate analysis of sociodemographic and clinical variables and mental health symptoms

Variables	Depression			Anxiety			Stress		
	With depressive symptoms	Without depressive symptoms	*P*	With anxiety symptoms	Without anxiety symptoms	*P*	With symptoms of stress	No symptoms of stress	*P*
Age (years) – mean± SD	29.0 ± 6.0	28.6 ± 5.9	0.617	29.2 ± 5.8	28.0 ± 6.2	0.162	29.1 ± 5.4	28.7 ± 6.1	0.640
Collection site – n(%)			<0.001			<0.001			<0.001
Alto MAÉ Health Centre	96 (70.6)	28 (30.1)		115 (76.2)	9 (11.5)		47 (82.5)	77 (44.8)	
Zimpeto Health Centre	40 (29.4)	65 (69.9)		36 (23.8)	69 (88.5)		10 (17.5)	95 (55.2)	
Who do you live with – n (%)			<0.001			0.012			0.003
With parents	32 (23.5)*	5 (5.4)		32 (21.2)*	5 (6.4)		15 (26.3) *	22 (12.8)	
With other relatives	8 (5.9)	4 (4.3)		7 (4.6)	5 (6.4)		5 (8.8)	7 (4.1)	
With partner	82 (60.3)	83 (89.2)*		99 (65.6)	66 (84.6)*		30 (52.6)	135 (78.5)*	
Alone	13 (9.6)*	1 (1.1)		12 (7.9)	2 (2.6)		7 (12.3) *	7 (4.1)	
Others	1 (0.7)	0 (0.0)		1 (0.7)	0 (0.0)		0 (0.0)	1 (0.6)	
With occupation (*n*=224) – n(%)	93 (69.4)	52 (57.8)	0.1	110 (74.8)	35 (45.5)	<0.001	39 (69.6)	106 (63.1)	0.467
Education (*n*=393) – n(%)			0.089			<0.001			0.599
None	1 (0.7)	0 (0.0)		1 (0.7)	0 (0.0)		0 (0.0)	1 (0.6)	
Primary	7 (5.1)	12 (12.9)		5 (3.3)	14 (17.9)*		4 (7.0)	15 (8.7)	
Basic	63 (46.3)	50 (53.8)		72 (47.7)	41 (52.6)		24 (42.1)	89 (51.7)	
Medium	49 (36.0)	23 (24.7)		53 (35.1)	19 (24.4)		22 (38.6)	50 (29.1)	
Bachelor’s degree	16 (11.8)	8 (8.6)		20 (13.2)	4 (5.1)		7 (12.3)	17 (9.9)	
Number of pregnancies –median (P25 – P75)	2 (1 – 3)	3 (2 – 3)	0.093	2 (1 – 3)	3 (2 – 4)	0.003	2 (1 – 3)	2.5 (2 – 3)	0.678
Hadmiscarriage/stillbirth – n(%)	31 (22.8)	25 (26.9)	0.582	33 (21.9)	23 (29.5)	0.266	14 (24.8)	42 (24.4)	1
Number currently alive – median (P25– P75)	1 (0 – 2)	1 (1 – 2)	0.112	1 (0 – 2)	2 (1 – 2)	0.002	1 (0 – 2)	1 (0 – 2)	0.929
Gestational trimester (*n*=228) –n(%)			0.234			<0.001			0.548
1st	30 (22.2)	30 (32.3)		27 (18.0)	33 (42.3)*		12 (21.4)	48 (27.9)	
2nd	70 (51.9)	43 (46.2)		79 (52.7)	34 (43.6)		31 (55.4)	82 (47.7)	
3rd	35 (25.9)	20 (21.5)		44 (29.3)	11 (14.1)		13 (23.2)	42 (24.4)	
Family planning – n(%)			<0.001			<0.001			<0.001
Yes	57 (41.9)	14 (15.1)		68 (45.0)	3 (3.8)		30 (52.6)	41 (23.8)	
No	79 (58.1)	79 (84.9)		83 (55.0)	75 (96.2)		27 (47.4)	131 (76.2)	
Religion – n(%)			0.078			<0.001			0.089
Atheist	0 (0.0)	1 (1.1)		0 (0.0)	1 (1.3)		0 (0.0)	1 (0.6)	
Católica	33 (24.3)	13 (14.0)		38 (25.2)*	8 (10.3)		16 (28.1)	30 (17.4)	
Muslim	20 (14.7)	7 (7.5)		26 (17.2)*	1 (1.3)		10 (17.5)	17 (9.9)	
Spiritist	3 (2.2)	5 (5.4)		1 (0.7)	7 (9.0)*		0 (0.0)	8 (4.7)	
Protestant	79 (58.1)	66 (71.0)		84 (55.6)	81 (78.2)*		30 (52.6)	115 (66.9)	
Other	1 (0.7)	1 (1.1)		2 (1.3)	0 (0.0)		1 (1.8)	1 (0.6)	
Marital status – n(%)			<0.001			<0.001			0.002
Married	31 (22.8)	14 (15.1)		38 (25.2)*	7 (9.0)		15 (26.3)	30 (17.4)	
Common-law marriage	26 (19.1)	44 (47.3)*		28 (18.5)	42 (53.8)*		7 (12.3)	63 (36.6) *	
Single	79 (58.1)*	35 (37.6)		85 (56.3)*	29 (37.2)		35 (61.4) *	79 (45.9)	
Monthly income –n(%)			0.067			0.15			0.602
< 10,000 mtn	87 (64.0)	70 (75.3)		100 (66.2)	57 (73.1)		38 (66.7)	119 (69.2)	
From 10,000 to15,000 mtn	41 (30.1)	15 (16.1)		42 (27.8)	14 (17.9)		16 (28.1)	40 (23.3)	
From 15,000 to20,000 mtn	6 (4.4)	4 (4.3)		7 (4.6)	3 (3.8)		1 (1.8)	9 (5.2)	
>20,000 mtn	2 (1.5)	4 (4.3)		2 (1.3)	4 (5.1)		2 (3.5)	4 (2.3)	
Devotes time to leisure activities – n(%)			0.119			0.058			0.010
Never	24 (17.6)	14 (15.1)		29 (19.2)	9 (11.5)		16 (28.1) *	22 (12.8)	
Rarely	78 (57.4)	47 (50.5)		87 (57.8)	38 (48.7)		32 (56.1)	93 (54.1)	
Occasionally	22 (16.2)	27 (29.0)		26 (17.2)	23 (29.5)		8 (14.0)	41 (23.8)	
Frequently	12 (8.8)	5 (5.4)		9 (6.0)	8 (10.3)		1 (1.8)	16 (9.3)	
Sleep quality (*n*=228) – n(%)			0.734			0.135			0.725
Satisfactory	103 (75.7)	67 (72.8)		117 (78.0)	53 (67.9)		44 (77.2)	126 (73.7)	
Unsatisfactory	33 (24.3)	25 (27.2)		33 (22.0)	25 (32.1)		13 (22.8)	45 (26.3)	
Undergoing long- term treatment – n(%)	18 (13.3)	9 (10.0)	0.586	22 (14.7)	5 (6.7)	0.128	9 (16.1)	18 (10.7)	0.398
If yes, what treatment (*n*=24) – n(%)			0.638			0.906			
ART	15 (88.2)	7 (100)		20 (90.9)	2 (100)		9 (100)	13 (86.7)	
Hypertension	1 (5.9)	0 (0.0)		1 (4.5)	0 (0.0)		0 (0.0)	1 (6.7)	
ART + Hypertension	1 (5.9)	0 (0.0)		1 (4.5)	0 (0.0)		0 (0.0)	1 (6.7)	
Receives follow-up care from a mentahealth professional – n(%)	8 (5.9)	5 (5.4)	1	11 (7.3)	2 (2.6)	0.228	7 (12.3)	6 (3.5)	0.020
Do you have anyrelatives who have had or are currently receiving mental health treatment or counselling? -n(%)	8 (5.9)	0	0.023	7 (4.7)	1 (1.3)	0.270	7 (12.3)	1 (0.6)	<0.001

* statistically significant association by the residuals test adjusted to 5% significance

*p*-values from Pearson chi-square or Fisher’s exact test

After adjustment in Poisson regression models with robust variance, several factors remained significantly associated with depressive symptoms. Women living with their parents had a 43% higher prevalence of depressive symptoms compared with those living with a partner (PR = 1.43; 95% CI: 1.17–1.76). Similarly, women living alone had a 60% higher prevalence of depressive symptoms (PR = 1.60; 95% CI: 1.31–1.96). Being married was also associated with increased prevalence, with married women presenting a 53% higher prevalence of depressive symptoms compared with those in common-law unions (PR = 1.53; 95% CI: 1.07–2.21). In addition, having a family history of mental illness was associated with a 63% higher prevalence of depressive symptoms (PR = 1.63; 95% CI: 1.32–2.01).

Anxiety symptoms were also associated with several sociodemographic and reproductive factors. Women living with their parents had a 38% higher prevalence of anxiety symptoms compared with those living with a partner (PR = 1.38; 95% CI: 1.12–1.70). Being married was associated with a 68% higher prevalence of anxiety symptoms compared with common-law unions (PR = 1.68; 95% CI: 1.26–2.23). Women with an occupation had a 35% higher prevalence of anxiety symptoms (PR = 1.35; 95% CI: 1.07–1.69). The use of family planning was associated with a 41% higher prevalence of anxiety symptoms (PR = 1.41; 95% CI: 1.21–1.64). Each additional pregnancy was associated with an 11% reduction in anxiety prevalence (PR = 0.89; 95% CI: 0.82–0.96). Having a family history of mental illness was associated with a 55% higher prevalence of anxiety symptoms (PR = 1.55; 95% CI: 1.26–1.91). Compared with Protestant women, Catholic and Muslim women showed higher prevalence of anxiety symptoms.

Stress symptoms were significantly associated with marital status, family planning use, and mental health history. Married women had a 41% higher prevalence of stress symptoms compared with those in common-law unions (PR = 1.41; 95% CI: 1.05–1.89). Women reporting the use of family planning methods had a 145% higher prevalence of stress symptoms compared with those who did not report family planning use (PR = 2.45; 95% CI: 1.60–3.76). Additionally, women receiving mental health follow-up showed a 177% higher prevalence of stress symptoms (PR = 2.77; 95% CI: 1.81–4.24). Given the limited number of events relative to the number of predictors in this model (EPV ≈ 4:1), these estimates should be considered exploratory and interpreted with particular caution (Table 4).

**Table 4:** Poisson regression models for factors associated with depression, anxiety, and stress symptoms

Depression DASS-21				Anxiety DASS-21				Stress DASS-21			
Variables	Prevalence Ratio	95% CI	*P*	Variables	Prevalence Ratio	95% CI	*P*	Variables	Prevalence Ratio	95% CI	*P*
Who they live with				Occupation	1.35	1.07 –1.69	0.01	Who do you live with?			
With parents	1.43	1.17 –1.76	<0.001	Religion				With parents	1.72	1.07 –2.77	0.025
With other relatives	1.15	0.74 –1.79	0.539	Atheist	*	*	*	With other relatives	1.32	0.71 –2.48	0.38
With partner (ref.)	1	-	-	Catholic	1.22	1.04 –1.44	0.015	With partner	1	-	-
Alone	1.6	1.31 –1.96	<0.001	Muslim	1.45	1.22 –1.72	<0.001	Single	2.47	1.30 –4.69	0.006
Others	1.48	1.24 –1.78	<0.001	Spiritist	0.23	0.03 –1.56	0.132	Others	*	*	*
Family planning	1.38	1.14 –1.68	0.001	Protestant	1	-	-	Family planning	2.45	1.60 –3.76	<0.001
Marital status				Other	1.2	0.97 –1.47	0.09	Receives follow- up care from a mental health professional	2.77	1.81 –4.24	<0.001
Married	1.53	1.07 –2.21	0.021	Family planning	1.41	1.21 –1.64	<0.001	Do you have any relatives who have seen or are seeing a mental health	3.87	2.62 –5.73	<0.001
Common-law marriage	1	-	-	Marital status							
Single	1.38	0.96 –1.97	0.079	Married	1.68	1.26 –2.23	<0.001				
Do you have any relatives who have seen or are seeing a mental health professional?											
				Common- law marriage (ref.)							
	1.63	1.32 –2.01	<0.001		1	-	-				
				Single	1.2	0.89 –1.62	0.223				
				Number of pregnancies	0.89	0.82 –0.96	0.002				

* risk could not be estimated due to insufficient number of cases

*PR* Prevalence ratio, *CI* Confidence interval. Ref.: reference category. Reference groups were living with partner (household composition), common-law union (marital status), Protestant (religion), no family planning use, no occupation, and no family history of mental illness

The DASS-21 demonstrated adequate internal consistency in the sample, with Cronbach’s α coefficients of 0.827 for depression, 0.799 for anxiety, and 0.813 for stress, indicating satisfactory psychometric reliability for use in pregnant women.

## Discussion

The findings of this study indicate a high prevalence of depressive, anxiety, and stress symptoms among pregnant women in Maputo, particularly at clinically significant levels [3, 16]. These findings are broadly consistent with global evidence indicating a high prevalence of perinatal psychological symptoms, especially in low- and middle-income countries. Substantial heterogeneity across studies has been reported, reflecting differences in measurement approaches, socioeconomic conditions, and health system contexts [17, 18]. Notably, the higher prevalence of anxiety symptoms compared with stress in our sample warrants a more nuanced interpretation. Although global estimates suggest relatively comparable prevalence of anxiety and depressive symptoms, our findings indicate a distinct pattern, possibly reflecting contextual and measurement-related differences [19–21].

From a measurement perspective, this discrepancy may be partially explained by differences in DASS-21 classification thresholds, as higher cut-off points for stress may reduce the proportion of cases identified at clinically significant levels [22]. Additionally, anxiety symptoms may be more sensitive to uncertainty, anticipatory concerns, and perceived lack of control during pregnancy, particularly in socioeconomically constrained environments. In contrast, stress symptoms, as operationalized by the instrument, may reflect more sustained physiological tension, which may be less frequently reported or differently interpreted in this context. Cultural differences in emotional expression and reporting may further contribute to this pattern [23]. The observed discrepancy suggests that the technical rigor of the DASS-21 cutoff scores may be underestimating stress, while anxiety stands out for better reflecting the uncertainties and socioeconomic vulnerabilities of pregnancy. These observations suggest that the phenomenon is not only clinical but also methodological and cultural, requiring an interpretation that considers how context shapes the expression and measurement of emotional distress.

Sociodemographic and clinical factors were significantly associated with psychological symptoms, supporting a biopsychosocial interpretation of maternal mental health [6–8]. Household composition was associated with depression and stress, with higher symptom prevalence among women living alone or with extended family, consistent with the hypothesis that social support may play an important role in maternal emotional well-being during pregnancy [24].

A family history of mental illness was associated with higher symptom prevalence across outcomes, consistent with evidence on familial aggregation of psychiatric disorders [20]. Marital status was also associated with psychological outcomes, with married women reporting higher symptom levels compared with those in common-law unions. While this finding contrasts with evidence suggesting a potential association between stable partnerships and better mental health outcomes, it must be interpreted with caution. As this study did not collect data on relationship quality, partner support, intimate partner violence, or household conflict, no definitive conclusions can be drawn regarding the effect of marriage itself. It is possible that, within this specific context, formal marriage may entail increased social, economic, and relational demands that contribute to greater psychological burden; however, the absence of data on relationship quality, partner support, and household dynamics precludes any definitive interpretation [5, 6, 23].

Occupational engagement was associated with higher anxiety in this sample, contrasting with evidence linking unemployment to poorer mental health outcomes during pregnancy [25, 26]. This divergence highlights the importance of contextual interpretation. Although 64.7% of participants were employed, the majority had a monthly income below approximately 157 USD, indicating that employment does not necessarily ensure economic security. Under these conditions, work may be associated with increased stress rather than reduced psychological distress, through greater exposure to financial strain, job insecurity, and competing domestic demands. These findings suggest that the quality and stability of employment, rather than employment status alone, may be important factors associated with maternal mental health outcomes [27, 28].

Mental health follow-up was associated with higher stress, likely reflecting reverse causation, whereby women with more severe symptoms are more likely to seek care. This association may also be influenced by healthcare contact bias, as women with greater exposure to health services may have increased symptom recognition and reporting. Structural limitations in prenatal care, including long waiting times and limited privacy, may further contribute to stress among those already engaged with the healthcare system [9, 10, 29].

The association between family planning and higher levels of anxiety and stress should be interpreted with caution. It is important to note that this study did not collect data on pregnancy intention, contraceptive failure, or reproductive decision-making; therefore, any mechanistic explanations involving these pathways cannot be tested within the current dataset. In low- and middle-income settings, contraceptive use does not necessarily reflect full reproductive autonomy, as it may coexist with inconsistent access to services and partner-related decision dynamics [30–32]. Reverse causation and healthcare contact bias may also contribute, as women more engaged with reproductive health services may be more likely to report symptoms [29], while residual confounding cannot be excluded.

An inverse association between parity and anxiety was observed, consistent with the hypothesis that prior maternal experience may be associated with enhanced coping strategies and greater self-confidence, potentially strengthening the perception of social support [33, 34]. Religious affiliation was also associated with higher anxiety prevalence, suggesting that doctrinal norms and moral expectations may, in some contexts, intensify maternal concerns; further qualitative research is warranted to explore these dynamics [23, 32].

The magnitude of the observed associations has important public health implications. Prevalence ratios of this magnitude suggest clinically meaningful differences in psychological vulnerability across subgroups. These findings may translate into increased demand for mental health services and highlight priority populations for targeted screening and intervention strategies within antenatal care programs. Interpreting these associations beyond statistical significance is essential for informing resource allocation and health system planning [10].

Differences between health centers highlight the influence of contextual factors. Alto Maé, an urban setting characterized by higher population density and greater exposure to urban stressors, showed a higher prevalence of psychological symptoms compared with peri-urban Zimpeto. These differences likely reflect variations in socioeconomic conditions, healthcare access, and structural stressors, which are known to shape mental health outcomes in low- and middle-income settings [9, 10]. These findings underscore the importance of situating epidemiological results within local health system and social contexts.

Regarding measurement properties, the Portuguese version of the DASS-21 demonstrated satisfactory internal consistency in this sample (Cronbach’s α > 0.70), consistent with findings from other settings [12, 35]. To our knowledge, the Portuguese version of the DASS-21 has been used in at least one prior study conducted in Mozambique [19], and is currently employed in clinical practice in the country, supporting its practical relevance in this context. However, formal cross-cultural psychometric validation in the Mozambican population has not yet been established. It is important to note that satisfactory internal consistency reflects the reliability of the instrument, but reliability alone does not establish construct validity. Cultural and linguistic differences in the conceptualisation and expression of psychological distress may affect how items are interpreted, potentially leading to under- or over-estimation of true prevalence and limiting direct comparisons with studies conducted in other settings. No diagnostic interviews were conducted; therefore, the results should be interpreted as indicators of symptom burden rather than confirmed clinical diagnoses.

Overall, these findings support a biopsychosocial interpretation of maternal mental health, whereby psychological outcomes during pregnancy arise from the interaction between structural conditions, social relationships, and individual factors [7, 8]. In this perspective, mental health extends beyond the presence or absence of symptoms, reflecting a multidimensional construct shaped by broader socioeconomic and healthcare system influences.

Limitations: Several limitations should be considered when interpreting these findings. First, the cross-sectional design precludes causal inference and does not allow temporal relationships between exposures and outcomes to be established.

Second, the study was conducted in only two primary health centres in urban and peri-urban areas of Maputo, which may limit the generalisability of the findings to other regions of Mozambique, particularly rural settings with different socioeconomic conditions and healthcare access. These contextual differences may also have influenced the composition of the study sample, as women attending these facilities may differ systematically from those with limited or no access to antenatal care.

Third, psychological symptoms were assessed using a screening instrument (DASS-21), and no diagnostic clinical interviews were conducted; therefore, the results should be interpreted as indicators of symptom burden rather than confirmed psychiatric diagnoses. Although the Portuguese version of the DASS-21 was used, formal cross-cultural validation in the Mozambican population has not yet been established, and linguistic or cultural differences in the interpretation of emotional distress may affect measurement validity and comparability.

Self-reported data may be subject to reporting bias, and unmeasured contextual and psychosocial variables may have acted as confounders. From a statistical perspective, although the included variables showed stable estimates across models, residual confounding cannot be excluded, as not all relevant factors may have been captured. The inclusion of multiple predictors also raises the possibility of model overfitting, particularly given the relatively small sample size in some subgroups. This concern is especially relevant for the stress outcome, where only 33 participants met the clinical threshold, resulting in an events-per-variable ratio of approximately 4:1 below the recommended minimum of 10:1. To address this, variable selection was restricted to those with *p* < 0.10 in bivariate analysis; nonetheless, the estimates from the stress model should be considered exploratory and interpreted with particular caution, pending validation in larger cohorts.

Despite these limitations, the high internal consistency observed (Cronbach’s α > 0.70) supports the reliability of the instrument. Future studies should prioritise longitudinal designs and the psychometric validation of the DASS-21 in Mozambique to improve causal inference and cultural validity.

## Conclusion

This study underscores the substantial psychological burden experienced by pregnant women in Maputo and highlights the complex interplay of socioeconomic, relational, and contextual factors shaping maternal mental health. The findings reinforce the need to address perinatal mental health not only as the presence or absence of symptoms but as a multidimensional process involving structural conditions, social support, and adaptive psychological functioning.

*Implications for clinical practice*: Systematic mental health screening should be integrated into routine antenatal care using culturally sensitive and psychometrically sound instruments, accompanied by clearly defined referral pathways to specialized services. Although the DASS-21 demonstrated satisfactory internal consistency in this sample, its implementation should be combined with clinical assessment and contextual sensitivity to linguistic and cultural nuances. Strengthening provider training and reducing structural barriers within prenatal services are essential to ensure that screening translates into effective support.

*Implications for public health policy*: Maternal mental health strategies in Mozambique must extend beyond individual-level interventions to address underlying social determinants. Economic insecurity, precarious employment conditions, and relational vulnerabilities were associated with psychological distress in this sample, highlighting the potential importance of structural responses. Policies aimed at improving social protection, income stability and access to integrated reproductive and mental health services are central to reducing maternal psychological vulnerability.

*Directions for future research*: Longitudinal studies are needed to clarify temporal relationships between identified factors and psychological symptoms. Further qualitative and mixed-method research should explore the contextual dynamics of family planning and religious influences on perinatal mental health. Additionally, future investigations should expand beyond a predominantly pathological focus to include factors potentially associated with resilience, high-quality social support, and adaptive coping processes. Research grounded in a biopsychosocial perspective should also consider positive psychological processes and health-promoting behaviours, such as physical activity and nutrition, in relation to maternal well-being.

These conclusions should be interpreted in light of certain limitations. The cross-sectional design precludes causal inference, and the absence of formal cultural validation of the DASS-21 in Mozambique may have influenced item interpretation. Nonetheless, the study provides important local evidence to inform clinical protocols, guide public health planning and support the development of culturally responsive maternal mental health research in the region.

## Data Availability

All data supporting the findings of this study are available within the paper and its Supplementary Information. The supplementary file includes the anonymised dataset used for the analyses, the coding of all study variables, and the complete output of the statistical models, including the Poisson regression with robust variance.

## Abbreviations

DASS: Depression, Anxiety and Stress Scale-21 items
PR: Prevalence ratio
CI: Confidence interval
CIBS FM&HCM: Committee of Bioethics for Health of the Faculty of Medicine/Central Hospital of Maputo
